# Practical Medicine

**Published:** 1857-05

**Authors:** 


### PRACTICAL MEDICINE.

1. ON THE TREATMENT OF THE NIGHT SWEATS OF CONSUMPTION. By *E. J. Coxe*, M.D., Visiting Physician to Charity Hospital, New Orleans, La.—"In the latter stages of consumption, of the many unfavorable symptoms frequently coexisting, no one is productive of greater distress to the sufferer than the exhausting night sweats, the difficulty or impossibility of arresting which is recognized by almost all of our standard authorities. Depending, in great measure, upon excessive debility, the result of the peculiar depressing influence of the disease upon the more important organs of the body, our main resource is to remove this, by the employment of a nourishing diet, mineral and vegetable tonics, and those external applications recommended by all practical writers.

"In directing attention to the employment and real value of certain remedies, which for some time I have used with marked effect in controlling and removing this most unpleasant symptom, it is proper to state, that in the wards of the Charity Hospital under my charge, as also in private practice, I have, during the past eighteen months, had opportunities of testing their success. Inasmuch as previously to employing them, all the usual remedies had been tried without permanent success, and as experience in a sufficient number of cases now authorizes a positive opinion, I cannot but regard it a duty to promulgate a knowledge of the fact.

"The remedies generally employed for the purpose of alleviating night sweats in consumption are the following: Small doses of opium, as found in Dover's powder, or compound kino powder; gallic or tannic acid, alone, or conjoined with morphia; sulphuric acid, as commonly used in elixir of vitriol, which last has been commended by the late Dr. Morton, used with sage tea, in the dose of a wine-glassful three times a day, or only at bed-time; a pill of sulphate of iron and alum, at bed-time, or more frequently during the day; a cold infusion of wild cherry bark. Dr. Theophilus Thompson, of London, speaks in high terms of the efficacy of four grains each of oxide of zinc and extract of hyoscyamus or henbane, and remarks that no remedy which he had employed had exercised so uniformly favorable an effect in moderating the night perspirations. He says that he had occasionally substituted the sulphate of zinc, in doses of two grains, with advantage, but, generally speaking, with less efficiency. Dr. T. observes, 'I am particularly anxious to direct your attention to the valuable properties of zinc, because the preparations of this metal have been disparaged by some writers of authority.' Although I had read Dr. T.'s work on Consumption previously, the practical importance of the above escaped me, and it was not until some time afterward, in perusing Dr. Barlow's Practice of Medicine, and disappointed with the want of power of the usual remedies, fully tried, that my attention was fixed upon the following sentence. 'It is in this stage that the night sweats are most troublesome, and against them there is no remedy equal to the combination of zinc and hyoscyamus.' The following is his formula: ℞. Zinci sulph., gr. i; Hyoscyami ext., gr. iv, to be made into one pill, and to be taken every night at bed-time. I would remark, that while this combination has succeeded by itself in most of the cases in which it was used, in a reasonably short time, in preventing the accustomed night sweats; in others, with the desire to improve the formula, if possible, different articles were added, varying the proportion of the essentials, in order to allow the exhibition of several pills during the day.

"My rule at present is to give the formula of Dr. B. at bed-time, and, several times during the day, a pill, composed of different ingredients—retaining, however, in some cases, a small portion of the zinc and hyoscyamus. In one instance, of recent occurrence, although a fatal issue could not be averted, the night sweats, which had long been profuse, were completely arrested by the pill at night and three others during the day, for many weeks before death. The number of cases, in which the night sweats of confirmed consumption have been entirely removed in from three to six days, at times earlier, and in one instance in private practice about two weeks, have been about thirty. In these cases the physical signs were well marked, and closely observed by many judges."—*Boston Med. and Surg. Journ.*, April 9, 1857.

2. TREATMENT OF POISONING BY STRYCHNINE. By *Marshall Hall*, F.R.S., &c.—"Judging from many experiments, I believe that strychnine destroys life in *three* ways:

"1. By inducing *laryngismus* and apnœa (or asphyxia).

"2. By inducing exhaustion of the nervous power, the effect of spasm and pain; and

"3. By a secondary asphyxia.

"The *first* object in the treatment is, of course, to get rid of the poison. Emetics must be given. But if these fail, the hopeful remedy is, to place the patient *prone*, and in the interval between the spasms, to tickle the fauces with a feather or other object.

"The *second*—the important remedy, is—*tracheotomy*. In my experiments, I gave the same poisonous dose of the acetate of strychnine to *each of two* dogs, and peformed tracheotomy in *one;* and left them undisturbed for the night. The one in which tracheotomy was performed, *lived;* the other infallibly *died!* Tracheotomy disarms laryngismus of danger—of its apnœa.

"The *third* remedy is—the *Ready Method*, with two objects: the first, to administer respiration as the remedy for the *effect* of the laryngismus, &c., apnœa or the suspension of respiration; the second, adding tickling to the fauces, again, to empty the stomach; a third may be, even when all spasm has ceased, to *continue* the alternate pronation and rotation, that is—respiration,—in the hope that life may be continued until the poison may be eliminated from the system, as well as mechanically regurgitated from the stomach."—*London Lancet*, February 28, 1857.

3. MR. STARTIN'S METHOD FOR THE PREVENTION OF PITTING IN SMALL-POX.—"The plan consists in applying the acetum cantharidis (P.L.) or any vesicating fluid,* by means of a camel-hair brush, to the apex of each spot or pustule of the disease, on all the exposed surfaces of the body, until blistering is evidenced by the whiteness of the skin in the parts subjected to the application, when the fluid producing it is to be washed off with water or thin arrow-root gruel.

* It will be observed, that this practice of vesication is the opposite to that recommended by the writers advocating nitrate of silver, who desire to avoid the blistering process.

"The vesication for each pustule should not be larger than the eighth or sixth of an inch in diameter, leaving intact the boundaries of the inflammation, excepting where the malady has become confluent, when the entire surface so affected should be vesicated.

"With respect to the best period of the eruption of small-pox for making this application, although between the fourth and eighth days should be preferred, the vesication has seemed to me to have been efficacious whenever it has been practised before the slough has formed, evidenced by the secretion of pus, which slough is at once the cause of the pitting, and the peculiar characteristic of this formidable malady.

"The only after-treatment in these cases consists in puncturing the blisters with a needle, in keeping the affected skin clean by means of arrow-root or rice gruel (avoiding soap of every kind), and in bathing the eruption several times a day by the aid of soft sponge or linen wetted with the following lotion: ℞. Sodæ biboracis, ℈j; Ammoniæ sesquicarbonatis, ℈j; Acidi hydrocyanici diluti, ʒj; Glycerini destillati, ℥ss; Aquæ rosæ ad ℥viij. M. Ft. lotio.

"The pain attending the application of the vesicating fluid is very slight and transient, disappearing almost simultaneously with the ablutions recommended, nor does the blistering add much to the disfigurement, while it relieves the pyrexia and cerebral symptoms, should they be present. The *rationale* of the benefits arising from the method appears to me to be comprised in John Hunter's observation, that 'if you can succeed in changing the character of an inflammation, you will often succeed in curing a specific disease;' which he calls 'the mode of cure by an irritation different from the disease;'* for he holds elsewhere, that 'no one disease can have two distinct and different critical inflammations.'"—*Medical Times and Gazette*, February 21, 1857.

4. ON THE MEDICINAL EFFECTS OF AMMONIA AND ITS PREPARATIONS.—At a late meeting of the London Medical Society, *Dr. Ogier Ward* read the following paper on the therapeutical effects of ammonia:

"Ammonia has never been considered to be a normal constituent of the blood, as its presence had not been detected except after death in cases of typhus, cholera, melæna, and other diseases of a putrid character, until Dr. Richardson's recent discovery that healthy blood owes its fluidity to the presence of ammonia, which is given off during its coagulation, which process may be arrested, and the fibrine re-dissolved, by the restoration of the alkali. An interesting inquiry here suggests itself: How does the ammonia escape from the body during the coagulation of the blood, and how is it retained in those instances in which the blood remains fluid after death? Assuming the truth of Dr. Richardson's views, the author examined and compared the therapeutic effects of the various preparations of ammonia; and he has found that, whether applied externally or taken inwardly, they possess in common the property of dissolving the proteine elements of the blood, whether in the vessels or effused into the tissues; and thus confirm the experiments of Dr. Richardson. The similarity in the effect of ammoniacal medicines is owing to their ready decomposition, the ammonia being separated, and forming the chief curative agent, though it is aided by the other substances

* Hunter on the Blood, vol. ii, page 131, et seq., vol. i, page 454.

originally combined with it. Thus its stimulant and solvent action is similar in kind, if not in degree, when used either externally or inwardly in the form of gas, liquor ammonia, or combined with carbonic acid, &c. From the utility of these preparations in the treatment of venomous bites and stings, inflammatory swellings, diphtheritis, croup, &c., we may suppose that they will be equally efficacious in urticaria, erythema nodosum, and erysipelas, in which there is an effusion of the fibrinous elements of the blood. In these and other inflammatory diseases and conditions, it is probable that the ammonia is carried out of the system in the form of urea or lithate of ammonia contemporaneously with the increase of fibrine in the blood; and that the benefit of the salts of ammonia in such cases is owing to their preventing or removing the effusion of fibrine from the inflamed parts. In this way, although the primary action of ammonia is stimulant, its remote effects are sedative or debilitant, as it not only arrests inflammatory action, but, by its resolvent and secernent power, carries the products of inflammation out of the system, and hence its utility in all active febrile complaints. It is to this attenuating property that its use as an antidote to drunkenness and to the stupor of opium is to be ascribed. Its stimulant powers are of use in poisoning by hydrocyanic acid, in the cold stage of ague, and in the retrocession of gout, rheumatism, and the exanthemata, as well as in syncope, hysteria, epilepsy, and convulsions. The hydrochlorate, which is the least easily decomposed, is probably the most useful of the salts of ammonia, as it not only possesses the stimulant, resolvent, secernent properties of the others, but owing to its combinations with chlorine, is endued with tonic powers, by which its prolonged use, unlike that of the other preparations, is attended with invigorating effects both to mind and body; and thus it forms an excellent substitute for mercury in cases where this medicine is inadmissible from its tendency to produce cachexia."—*Lancet*, Feb. 4, 1857.

5. TREATMENT OF APNŒA (OR ASPHYXIA). By *Marshall Hall, M.D.*, NEW RULES, OR THE "READY METHOD."—*Lose not a moment of time;* treat the patient ON THE SPOT, *in the open air*, exposing the face and chest freely to the breeze, except in cold weather; then—

I. *To Excite Respiration.*

Place the patient gently, and for a moment, on the face, to allow any fluids to flow out of the mouth:

Then raise the patient into the sitting posture, and endeaver to *excite* respiration;

1. By irritating the nostrils by *snuff, hartshorn*, &c.
2. By irritating the fauces by a *feather*, &c.
3. By dashing *hot* and *cold* water *alternately* on the face and chest.

If these means fail—

II. *To Imitate Respiration.*

Replace the patient on his face, his arms under his forehead, and—

1. Turn the body gradually, but completely, on the *side*, and *a little more*, and then again on the face, alternately;
2. When replaced, apply pressure along the back and ribs, and then remove it, and proceed as before;

3. Let these measures be repeated gently, deliberately, but efficiently, and perseveringly, *sixteen* times in the minute *only*.

III. *Continuing* these measures, rub all the limbs upwards, making firm pressure, *energetically*.

Replace the wet clothes by such other covering, &c., as can be procured.

*Omit the warm bath until respiration be re-established.*

Here the one idea is, the *throat* being cleared, to *excite* or *induce* respiration.

But to these "*Rules*," I must add a paragraph from the same pamphlet on the special subject of the apnœa (or asphyxia) of the still-born:

"In treating the still-born, the great object is, as usual, to *excite* respiration; this is most efficiently done by plunging it into (not a *warm*, but) a *hot* and *cold* bath alternately. This means of exciting respiration was first put to the test of experiment, at my suggestion, by my late deeply regretted friend, Mr. Henry Smith.

"The just temperatures of these baths have not yet been determined. I would suggest from 50° to 60° Fahr. for the cold, and from 98° to 102° for the hot bath. The immersion should be *momentary*; the alternation *quick*."

Experiments innumerable have demonstrated that if the subject be laid *prone*, and pressure be made briskly on the back, there is good *ex*piration; and that if the pressure be removed, and the body be turned *on its side and a little more*, there is good *in*spiration; that if this pronation and pressure, and this removal of the pressure and rotation be instituted *alternately*, there is good *Respiration*. The measures, then, are the true and instant remedy for apnœa (or asphyxia).

The whole may be reduced to the space of a nutshell, and to the form of a *quasi* syllogism:

Pronation and Rotation induce ample respiration:

Respiration is *the* remedy for apnœa (or asphyxia):

Therefore—Pronation and Rotation are *the* remedies for apnœa (or asphyxia.)

But not a *moment*, far less "a few minutes," is to be lost; "cita mors venit, aut victoria læta." Our motto must be "*principiis* obsta;" or, in good old English, act wisely, "*instantly*," "on the spot." There must be absolutely *no* delay; no removal, no warm-bath, no galvanism; no tardy and inculpatory use of a new mode of treatment, said to be on its *trial*, but already *proved* to be effectual by the profession. Nothing, in a word, short of the *instant* "Ready Method" *on the spot*. This is the only mode of doing justice to the important subject.

Being well imbued with this one idea, let any and every subsidiary measure, such as *excitants* of respiration, the douche of hot and cold water, excitation of the nostrils, the throat, the face, the general surface; but especially pressure applied with brisk movement of the limbs upwards, to *promote* the circulation and warmth, be superadded.

And let our proceedings be marked by fairness. Apnœa soon becomes asphyxia indeed; and it is still doubtful whether any case in which the heart has ceased to beat, and this occurs in a very "few minutes," has been restored.

This fact is indeed the great argument for the instant use of our best measures.

Perhaps the still-born infant presents us with the fairest and most frequent opportunities for the due administration of remedies and observations of their true effects. Already *fourteen* cases of success are recorded. Let us carefully avail ourselves of these opportunities.

It must be remembered that there are *three* stages of asphyxia: the first, that in which respiration may be *excited;* the second, that in which respiration cannot be excited, but may be beneficially *imitated;* these are both most appropriately designated *apnœa;* the third, true *asphyxia,* that in which not even induced respiration, or all that is comprised in the "Ready Method," should be expected to do any good. But in *all,* where there is a hope, this hope is realized by—*Pronation* and *Rotation,* with *Pressure* duly applied, and removed; but instituted *instantly,* and *on the spot.*—*Lancet,* Feb. 14, 1857.

6. Treatment of Hooping-Cough by Enemata of Assafœtida.—M. Ancelon, after passing in review, in the Annales de la Flandre, other modes of treating hooping-cough, on which occasion he greatly underrates the value of Belladonna, among the narcotics, indicates his decided preference for assafœtida given per anum. He declares it to be a reliable and an heroic remedy, in the second and third periods of the disease. Little can be expected from it in the first period. Much of its efficacy will depend on the dose and mode of administration. To infants eighteen months to two years old, three injections should be directed, each of them containing, in the smallest possible quantity of vehicle, fifteen grains of assafœtida and two drops of Sydenham's Liquid Laudanum. The first of these is to be administered in the evening; the second on the following morning; and the third in the evening, from twelve to fifteen hours after the second enema. M. Ancelon believes that in the second period the disease can be at once and entirely arrested. The third period presents greater difficulties and requires longer persistence in the use of the remedy, which must be continued twelve to fifteen days consecutively, while we associate with it as adjuvant, frictions on the skin with dry flannel or that which has imbibed oil of turpentine. M. Ancelon directs attention to the fact of which he has satisfied himself, that neither opium nor assafœtida, when given separately, produce any decided effect. Having had, as he believed, a certain remedy at hand, he was not disposed to make trials of sulphuric acid or of alum.

[Assafœtida has long been a familiar remedy in this country, in hooping-cough, at least as far as the teachings and writings of Dr. Chapman extended. With him this medicine was a favorite in this disease, and especially in combination with one of the alkalies; but although no stranger to its use as an enema, he did not so formally and emphatically recommend it in this way as is now done by M. Anceldon.

Some salutary hints are furnished by this writer in the impromptu treatment of two infants in violent paroxysms of hooping-cough. The first was six months old, and when visited it was in a room heated to an extreme degree, and a prey to an epileptiform fit of coughing. Snatching it up instinctively in his arms, M. Ancelon took it to the window, which he opened; the season, that of winter, was remarkable for its coldness, and he exposed the little being to the air of a temperature of sixteen degrees below freezing-point. A deep inspiration and some easy coughing soon indicated a cessation of the fit. On the second occasion, the same good result was obtained by dashing cold water on the face of the little sufferer. These, the author judiciously adds, are extreme palliatives, which give time for recourse to regular treatment.]

